# Cortical similarity networks in the rat brain: Postnatal development and sensitivity to early life stress

**DOI:** 10.1162/NETN.a.546

**Published:** 2026-04-22

**Authors:** Rachel L. Smith, Stephen J. Sawiak, Lena Dorfschmidt, Ethan G. Dutcher, Jolyon A. Jones, Joel D. Hahn, Olaf Sporns, Larry W. Swanson, Paul A. Taylor, Daniel R. Glen, Jeffrey W. Dalley, Francis J. McMahon, Armin Raznahan, Petra E. Vértes, Edward T. Bullmore

**Affiliations:** Department of Psychiatry, University of Cambridge, Cambridge, UK; Human Genetics Branch, National Institute of Mental Health, Bethesda, MD, USA; Behavioural and Clinical Neuroscience Institute, University of Cambridge, Downing Site, Cambridge, UK; Department of Physiology, Development and Neuroscience, University of Cambridge, Cambridge, UK; Department of Psychology, University of Cambridge, Cambridge, UK; Department of Biological Sciences, University of Southern California, Los Angeles, CA, USA; Indiana University Network Science Institute, Indiana University, Bloomington, IN, USA; Department of Psychological and Brain Sciences, Indiana University, Bloomington, IN, USA; Scientific and Statistical Computing Core, National Institute of Mental Health, NIH, Bethesda, MD, USA

**Keywords:** Connectome, Architectome, Micro-structural MRI, Translational neuroscience, Structural similarity

## Abstract

Network models are a key tool in human neuroscience, and translation into animal models is essential for interrogating mechanistic drivers of network organization. Using magnetic resonance imaging (MRI), we present the first in vivo network representation of the individual rat brain, a key animal model in neuroscience. We measured magnetization transfer ratio (MTR) at each of 53 distinct cortical areas and estimated a cortical similarity network for each scan across two independent cohorts. We characterized normative network development in rats scanned repeatedly between postnatal days 20 (weanling) and 290 (mid-adulthood; *N* = 47) and then contrasted these findings with a cohort exposed to early life stress (repeated maternal separation [RMS]; *N* = 40). The normative rat cortical similarity network exhibited biologically meaningful organization, consistent with cytoarchitectonic and tract-tracing data, and displayed complex topological features. Developmental analyses revealed increasing interregional similarity during early postnatal and adolescent periods, followed by divergence in mid-adulthood, particularly within fronto-hippocampal systems. RMS disrupted these trajectories, especially between frontal and parahippocampal regions that were also most dynamic during development and aging. These findings introduce a new network-based methodology for studying cortical organization in a model organism, providing a translational framework to understand how environmental risk factors alter brain network development.

## Author Summary

Structural similarity analysis has recently emerged as a method to map systems-level cortical organization using magnetic resonance imaging. While this technique has been primarily applied in humans, translation to animal models is essential for investigating the mechanistic drivers of network architecture and dynamics. Here, we present the first in vivo cortical similarity network of the individual rat brain, constructed using MTR measured across 53 cortical regions. We demonstrate that these networks exhibit a biologically meaningful organization and reflect tract-tracing and cytoarchitectonic data. Longitudinal analysis from weaning to adulthood revealed that fronto-hippocampal systems are highly dynamic during development and specifically vulnerable to early life stress. This work establishes a translational framework for quantifying how environmental risk factors shape brain network development.

## INTRODUCTION

Altered trajectories of brain development are a major risk factor for psychiatric and neurodevelopmental disorders (Lupien et al., 2009; Paus et al., 2008; Sánchez et al., 2001). However, the biological mechanisms that govern brain development—and how they are shaped by life experiences—remain incompletely understood. Tools that capture dynamic, systems-level changes in neuroarchitecture can advance our insight into these processes. Network models of macroscale brain organization, particularly those constructed using noninvasive neuroimaging, provide a key framework for characterizing distributed brain structure and function (Bullmore & Sporns, 2009, 2012; Fornito et al., 2015; van den Heuvel & Sporns, 2011, 2013). By quantifying interregional relationships, these models reveal the organization and maturation of brain systems across postnatal development and in response to experience.

Structural similarity analysis is an emerging approach for constructing individualized brain networks using magnetic resonance imaging (MRI; Li et al., 2017; Sebenius et al., 2023; Seidlitz et al., 2018). These methods estimate interregional similarity using MRI-derived morphometric features (Sebenius et al., 2025), resulting in networks in which nodes represent cortical areas and edges reflect the strength of structural similarity between them. A recent implementation, Morphometric INverse Divergence (MIND), quantifies similarity between voxel- or vertex-level distributions of MRI features (Sebenius et al., 2023). Conceptually, MIND analysis captures cortical patterns of similarity between areas at the macro scale of MRI that reflect their cyto- or myelo-architectonic similarity (high MIND), or differentiation (low MIND; Sebenius et al., 2025). Regions that are more architectonically similar are in turn more likely to share axonal connectivity (García-Cabezas et al., 2019; Goulas et al., 2014); thus, MIND networks can also serve as an approximation of the cortical “connectome” or axonal wiring diagram (Sebenius et al., 2025). MIND and related networks are reproducible (Sebenius et al., 2023; Seidlitz et al., 2018), developmentally sensitive (Dorfschmidt et al., 2024; Fenchel et al., 2020; Ruan et al., 2023; Sebenius et al., 2023; Wu et al., 2023), and responsive to environmental exposures (González-García et al., 2023; Hettwer et al., 2024; Tian et al., 2021; Xiao et al., 2023). MIND networks also show clinical relevance (Cao et al., 2023; Homan et al., 2019; Li et al., 2019; Lisowska & Rekik, 2019; Mahjoub et al., 2018; Zhang et al., 2020), significant heritability (Sebenius et al., 2023), and associations with gene expression (Morgan et al., 2019; Seidlitz et al., 2020).

Despite these advantages, structural similarity networks have been almost exclusively studied in humans, limiting their utility for testing causal mechanisms. Animal models are essential for addressing this gap, enabling experimental manipulation and brain tissue access. Structural MRI-based network analyses have been extensively applied in other small animals, including mice (Allan Johnson et al., 2019; Coletta et al., 2020; Pagani et al., 2016; Yee et al., 2018) and rabbits (Lim et al., 2015), providing valuable insights into macroscale brain architecture and revealing global organizational principles comparable to those observed in humans. However, although rats offer distinct advantages—including suitability for high-throughput experiments and ethologically rich, human-relevant behaviors (Bryda, 2013; Ellenbroek & Youn, 2016)—comparatively little is known about network representations of the rat brain. Existing data, primarily derived from expertly curated tract-tracing studies (Swanson et al., 2017, 2019, 2020, 2022, 2024a, 2024b), suggest that the rat connectome exhibits complex topology (Swanson et al., 2024b) akin to that in humans (Bullmore & Sporns, 2009) and mice (Rubinov et al., 2015). However, these data represent the composite rat brain and thus do not support individual-level analyses.

Here, we extend the MIND framework to rats, enabling construction of individual-level cortical similarity networks from in vivo MRI and providing a tool to examine how early life environments shape brain organization. We focus on magnetization transfer ratio (MTR), the most myelin-sensitive magnetic resonance proxy for cortical myelination (Mancini et al., 2020). Myelination is both developmentally dynamic (Downes & Mullins, 2014; Hamano et al., 1998; Mengler et al., 2014) and environmentally sensitive in rats (Abraham et al., 2023; Bass et al., 1970; Breton et al., 2021; Han et al., 2022; Krigman & Hogan, 1976; Long et al., 2021), but underexplored in rat MRI studies (Bai et al., 1996).

**Figure 1.**
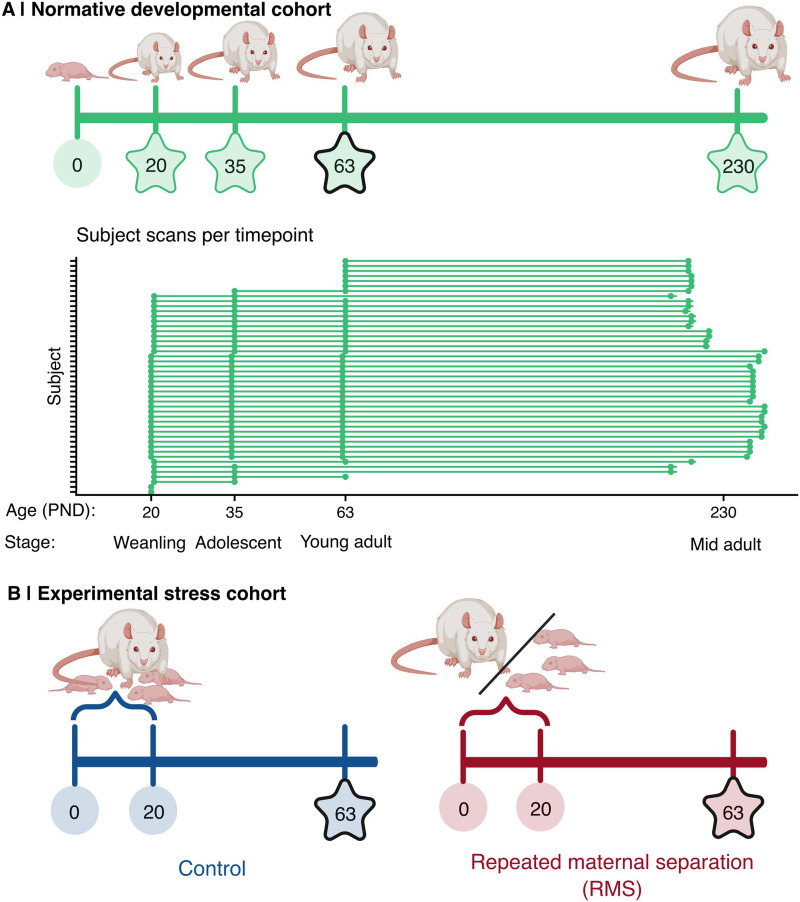
Two independent experimental cohorts to assess reliability and validity of rat MRI similarity networks as measures of developmental and stress-related changes in cortical microstructural networks. (A) Top: Study design for the normative developmental cohort: *N* = 47 male Lister Hooded rats were reared normally and had brain MRI scanning at PND 20 (*N* = 40), PND 35 (*N* = 38), PND 63 (*N* = 42), and within a few days of PND 230 (*N* = 43). All MRI timepoints are indicated by a star; PND 63 is outlined in black, as it marks the timepoint used to construct the normative network. Bottom: Scans per subject. Each tick on the *y*-axis represents a subject, while the *x*-axis shows age. Each point represents a scan; repeated scans of the same rat are connected by a line. (B) Study design for the experimental stress cohorts. *N* = 21 Lister Hooded pups were stressed by RMS; that is, for 6 hours per day every day from PND 5 to PND 19, preweaning pups were separated from their dam. A control group of *N* = 19 pups was reared normally. All animals completed MRI scanning at PND 63 as young adults. As with panel A, MRI timepoints are indicated by stars, and the primary comparison timepoint (PND 63) is outlined in black. The figure was created in https://BioRender.com.

We applied this method to two rat neuroimaging datasets: a longitudinal normative developmental cohort of males scanned across postnatal development and aging (Figure 1A), and an experimental stress cohort of males and females exposed to repeated maternal separation (RMS) as a model of early life stress (Figure 1B). We demonstrate that MIND networks reflect biologically meaningful features of cortical organization that track age- and experience-related changes in network architecture. These findings offer a novel network approach for linking early life experiences to brain development.

## MATERIALS AND METHODS

### Experimental Design

Preexisting structural MRI data from two independent cohorts, published in Jones et al. (2024) and Dutcher et al. (2023), were used to assess network-level changes that occur during development and in response to stress:

1.  Male normative developmental cohort (Jones et al., 2024; Figure 1A).

Male Lister Hooded rats (*N* = 47) were kept on a reverse light/dark cycle with red light on from 7:30 am to 7:30 pm and white light for the other half of the daily cycle. Rats underwent MRI scanning and weaning on postnatal day (PND) 20 or 21 (*N* = 40; here referred to as PND 20). Rats were scanned again at PND 35 (*N* = 38), PND 63 (*N* = 41), and once between PND 212–244 (*N* = 43; here referred to as PND 230). Note that not all 47 rats were scanned at every timepoint, which accounts for the variation in *N* reported across ages. Figure 1 shows the number of observations per animal in the male normative developmental cohort (hence referred to as the normative developmental cohort), with 32 animals (68% of total) having scans at each of the four timepoints from PND 20 (postweaning) to PND 230 (aging adult). Experiments were carried out in accordance with the (UK Animals) Scientific Procedures Act (1986) under UK Home Office project licenses (PPL 70/7587 & PPL 70/8072) and were approved by the University of Cambridge Ethics Committee.

2.  Experimental stress cohort (Dutcher et al., 2023; Figure 1B).

Pregnant Lister Hooded rats (*N* = 14) were purchased from Envigo (Blackthorn, UK). Litters were delivered by spontaneous partum on gestational days 22–24. Within 3 days of birth, litter size was adjusted to four to six pups, with each litter consisting of two female and two male pups (with the exception of one litter with four males and two females). If two litters were born within 24 hours of one another (the case for 10 litters in total), pups were mixed between the litters. After litter size adjustment, litters were allocated alternately by birth time to either the RMS condition (*N* = 30 pups: 14 female, 16 male) or the control condition (*N* = 28 pups: 14 female, 14 male). PND 0 was defined as the day of delivery. Body weight was measured weekly starting at PND 20. Lights were on from 21:00 to 09:00. Experiments were conducted on Project License PA9FBFA9F, in accordance with the UK Animals (Scientific Procedures) Act 1986 Amendment Regulations 2012, the EU legislation on the protection of animals used for scientific purposes (Directive 2010/63/EU), and the GSK Policy on the Care, Welfare and Treatment of Animals, following ethical review by the University of Cambridge Animal Welfare and Ethical Review Body.

From PND 5–19 (inclusive), pups from RMS litters were separated from their dam for 6 hours a day, starting between 11:00 am and 12:30 pm. During separation, dams remained in their home cages while pups were taken to a different room and placed together inside a ventilated cabinet. One centimeter of bedding was provided, and the temperature at the surface of the bedding was kept between 30 °C and 35 °C through warming of the air and use of an electric heat pad. Control pups were subject only to normal animal facility rearing. Following PND 20, pups from both groups were weaned and housed in same-sex pairs at PND 20 and left undisturbed until early adulthood except for weighing and once-weekly cage changes. All animals then underwent MRI scanning at PND 63, corresponding to young adulthood—a developmental stage sufficiently distant from the early maternal separation procedure to assess more persistent effects of early life stress in the context of mature cortical myelination.

### MRI Acquisition

For both cohorts, high-resolution MRI was performed on a 9.4 T horizontal bore MRI system (Bruker BioSpec 94/20; Bruker Ltd.). Images were acquired under isoflurane anesthesia using the manufacturer-supplied rat brain array coil with the rat in a prone position. Structural images were obtained based on a 3D multi-gradient echo sequence (TR/TE 25/2.4 ms with 6 echo images spaced by 2.1 ms, flip angle 6° with radio frequency spoiling of 117°). The field of view was 30.72 × 25.6 × 20.48 mm^3^ with a matrix of 192 × 160 × 160 yielding isotropic resolution of 160 *μ*m with a total scan time of 6 min 36 sec with zero-filling acceleration (25% in the readout direction; 20% in each phase encoding direction). Magnetization transfer (MT) pulses (10 *μ*T, 2 kHz off-resonance) were applied within each repetition to enhance gray-white matter contrast. Postreconstruction, images from each echo were averaged after weighting each by its mean signal.

Throughout all scanning procedures, rats were anesthetized with isoflurane (1%–2% in 1 L/min O2: air 1:4). Respiratory rate, oxygen saturation, and pulse rate (SA Instruments; Stony Brook, NY) were measured with anesthetic dose rates adjusted to ensure readings remained within a physiological range. Body temperature was measured and regulated with a rectal probe and heated water system to 36–37°C.

### Image Registration

Structural MRI image preprocessing was performed using the Analysis of Functional NeuroImages (AFNI) software package version AFNI_24.2.03 (Cox, 1996). Briefly, MT images for each rat were first deobliqued, spatially oriented, and translated to have spatial overlap with the Waxholm Space (WHS) reference template (Kleven et al., 2023). This space was selected due to its alignment with gold standard histological rat brain atlases (Paxinos & Watson, 2006; Swanson, 2018) and the atlas’s parcellation granularity (*N* = 222 regions). The @animal_warper command (Jung et al., 2021) was then used to nonlinearly align all MT images to the WHS template. Because some input datasets were notably smaller than the WHS standard template, the -init_scale option was added and used to increase the search space of the registration algorithm, scaled to the relative size ratio of the input scan to the template. Quality control image outputs for each scan (produced by @animal_warper) were then manually reviewed; for any images that were not successfully registered, @animal_warper was run again with the -init_scale parameter altered to better approximate the size ratio. All image registration scripts are available with this publication.

### MTR Calculation

MTR was calculated on a per-voxel basis for each scan in native space. First, native scans were scaled according to the receiver gain parameter used in the scan acquisition protocols. The scaled MT and proton density (PD) scans in native space were then used to calculate MTR according to the equation (PD − MT) / PD. Both steps were executed using 3dcalc in AFNI (Cox, 1996).

Manual evaluation of MTR data quality resulted in the exclusion of seven scans, typically because of motion artifacts and particularly at the earlier timepoints. Example MT images and their quality control images are shown in Supporting Information Figure S1. The analyzable MRI datasets available following preprocessing and quality control of the two cohorts are summarized in Table 1.

**Table 1.** Number of MRI scans included in the study analyses following quality control

	**PND**	***N***
**Normative developmental cohort**		
	20	40
	35	38
	63	41
	230	43
**Experimental stress cohort**		
RMS	63	21
Normal maternal rearing (control)	63	19

*N* represents the number of unique individuals scanned at each timepoint within each cohort.

### MIND Network Calculation

MIND networks estimate structural similarity from MRI data (Sebenius et al., 2023). Briefly, cortical regions are represented by a distribution of structural MRI features sampled at many points within the region, in this case, at each voxel. The MIND similarity between each pair of regions is then calculated using the Kullback–Leibler (KL) divergence between their feature distributions. The rationale for the structural features used in this work is detailed in the Supporting Information.

#### Input data generation.

Each MTR image was aligned with the WHS atlas in the native space of the respective MT scan (the scan with the highest contrast), so that each voxel in the MTR scan was labeled with a region of interest based on voxel assignment output from the registration pipeline. For each scan, a two-column comma-separate values (CSV) file was generated, in which the first column “Label” was the region of interest and the second column (“MTR”) was the value for the corresponding voxel in the MTR scan. For cortical MIND calculation, the input CSV was filtered to contain only voxels belonging to regions under the “Cerebral cortex” hierarchical level of the WHS atlas (which excludes olfactory bulb regions).

#### MIND network construction.

MIND networks were constructed for each scan by calculating the KL divergence between pairwise combinations of regional MTR profiles using the MIND toolkit (https://github.com/isebenius/MIND). The MIND algorithm used can be sensitive to differences in number of datapoints (in other words, region size). To balance this, we estimated KL divergence for each pair of regions by estimating the Gaussian distribution of MTR voxel values and then randomly sampling the same number of voxels from each distribution. In the main text, we chose *N* = 5,000 resamples; however, the network was not sensitive to the number of resamples chosen (Supporting Information Figure S2).

#### MIND network phenotypes.

In network neuroscience, topography refers to the spatial layout of brain regions, while topology refers to how those regions are interconnected. Here, we focus on the topological structure of the normative cortical MIND network.

Edge weight and nodal strength (or weighted degree) were considered features of interest for downstream analyses. Edge weights were calculated as *w* = 1 / (1 + KL), per the MIND toolkit (Sebenius et al., 2023). Nodal strength was calculated as the sum of all edge weights for a given region; hubs were defined as the 10 regions with the highest nodal strength. The rich club coefficient (*Φ*), a network measure that quantifies the tendency of hubs to be more densely interconnected compared with lower-strength nodes (Fornito et al., 2016a; van den Heuvel & Sporns, 2011), was calculated for the normative network as follows:

$$\phi_{w}\left(s\right)=\frac{W_{>s}}{W_{\text{max},>s}}$$ (1)

where

• *W*_>*s*_ = sum of edge weights among nodes with strength greater than *s* (i.e., hubs)
• *W*_*max*, >*s*_ = sum of the strongest edges in the network

### Edge Distance Calculation

The midline of the WHS atlas was determined using the AFNI function 3dcalc to separate the left and right hemispheres. To approximate the center of each WHS region of interest, the AFNI function 3dCM -Icent (Cox, 1996) was then used. The Euclidean distance between pairwise regional centers was then calculated as their edge length.

### Rat Atlas Mapping

We mapped the WHS atlas into Brain Maps 4.0 (BM4) atlas space (Swanson, 2018), Zilles atlas space (Zilles, 2012), and Allen Mouse Brain Atlas (AMBA) space (Lein et al., 2007) to compare the MIND similarity network to cortical tract-tracing data (Swanson et al., 2017, 2024b), cortical type (García-Cabezas et al., 2023), and mouse spatial transcriptomic expression (Yao et al., 2023), respectively. We did so first by aligning cortical regions based on nomenclature. However, not all regions maintained consistent nomenclature across atlases, so we also visually inspected anatomical alignment of regions using the WHS EBRAINS online resource (https://www.ebrains.eu/tools/rat-brain), BM4 atlas maps (https://sites.google.com/view/the-neurome-project/brain-maps), Zilles atlas cortical maps in stereotaxic coordinates (available for download at https://link.springer.com/book/10.1007/978-3-642-70573-1), and AMBA online interactive atlas viewer (https://atlas.brain-map.org/atlas?atlas=1). Briefly, we identified the position of each region in the reference (BM4, Zilles, or AMBA) atlas maps and then panned through the WHS atlas using the EBRAINS tool to approximate the same coronal slice and identify what region most closely aligned with the reference anatomical position. We also considered relative positioning of surrounding regions to determine this alignment. All atlas mappings are provided as resources in Supporting Information Tables S1A–C.

In this work, the BM4 atlas was used as the reference space for the tract-tracing comparison, the Zilles atlas was used as the reference space for the cortical type comparison, and the AMBA atlas provided reference space for the mouse transcriptomics comparison. If multiple WHS atlas subdivisions comprised a single reference atlas region, the median across these subdivisions was taken as the MIND edge weight for that region.

### Tract-Tracing Jaccard Index Calculation

To convert the cortical tract-tracing matrix (Swanson et al., 2017, 2024b) into a similarity network, the Jaccard index between each pairwise combination of regions was calculated. The set for a given region *a* was defined as the ordinal tract-tracing weight with each other region. The Jaccard index *J* between two regions *a* and *b* was then calculated as the intersection of their sets divided by the union as follows:

$$J\left(a,b\right)=\frac{|A\cap B|}{|A\cup B|}$$ (2)

### Cortex Type Network Thresholding

WHS cortical regions were categorized by their cortical type using the data presented in García-Cabezas et al. (2023) and grouped according to whether they are part of the archicortical allocortex or mesocortex (agranular or dysgranular subdivisions). Paleocortical and eulaminate regions were excluded from this analysis due to the very small number of constituent regions defined by the atlas. Each MIND edge was then defined as “intraclass” or “interclass” based on whether both regions were part of the same cortex type or not, respectively. To assess the extent to which top-weighted MIND edges consisted of two regions within the same cortex type, the normative MIND network was thresholded across densities (comprising 0%–10% of top-weighted edges), and the percentage of intraclass edges was calculated.

### Statistical Analyses

#### Correlation calculation.

When testing alignment between MIND networks and other neurobiological features, either Pearson’s or Spearman’s correlation was used. Pearson’s correlation (*r*) was applied to continuous, same-scale measures that exhibited approximately linear relationships (as visualized on scatterplots), including MIND edge weight versus interregional distance and between-cohort comparisons of MIND edge weights. Spearman’s rank correlation (*ρ*) was used for comparisons involving rank-ordered or sparse data with nonlinear relationships, such as tract-tracing and transcriptomic alignments. Normality testing was not used as a basis for this decision due to its high sensitivity at large sample sizes (Ghasemi & Zahediasl, 2012). As a sensitivity analysis, both Pearson’s and Spearman’s correlation coefficients for each relationship are reported in the Supporting Information Table S2.

#### Null network generation.

Ten thousand null networks were generated to assess whether normative MIND network alignment with tract-tracing similarity and cortical type were greater than would be expected by chance. To do so, all MIND network edges were classified into three evenly sized bins based on distance: proximal, intermediate, and distal. Edge weights within each bin were then reshuffled to generate a null network that preserved distance structure. The tract-tracing and cortical type analyses were repeated for each null network to generate a null distribution for comparison of each analysis.

#### Developmental slope.

Changes that occurred in edge weight (*w*) and nodal strength (*s*) during normative development (Δ*w*_dev_; Δ*s*_dev_) and aging (Δ*w*_age_; Δ*s*_age_) were quantified by calculating the linear gradient or slope of age-related change in each period. Early development was defined as PND 20 to PND 35, as the highest proportion of change occurred here, and aging was defined as PND 63 to PND 230. For each region, a linear mixed effects model was fit with the normalized strength as the dependent variable, continuous age and total brain volume as fixed effects, and each individual rat as a random effect (TBV = total brain volume):

$$\text{normalized strength}\sim \beta_{1}age+\beta_{2}TBV+\left(1|\text{subject}\right)$$ (3A)

Edge-level analyses were run using coarse-grained systems labels for interpretability. In this case, region of interest (ROI)-level edges were also included as a random effect:

$$\text{normalized weight}\sim \beta_{1}age+\beta_{2}TBV+\left(1|\text{subject}\right)+\left(1|ROI\;\text{edge}\right)$$ (3B)

The coefficient for age, *β*_1_, was estimated as the slope for that region within the given period. For visualization and comparison between epochs, we used the associated *t*-statistic (denoted as Δ*w* for edges and Δ*s* for nodal strength) to represent the standardized effect of age. Due to the inherent ambiguity in estimating degrees of freedom for mixed-effects models, we report *t*-statistics to indicate the significance of fixed effects.

#### RMS-control effect size.

Case–control analysis at PND 63 was used to identify changes that occurred in response to RMS and were measurable in young adulthood. For each region, the following model was used:

$$\text{normalized strength}\sim \beta_{1}\text{group}+\beta_{2}sex+\beta_{3}age+\beta_{4}TBV$$ (4A)

As with development, case–control edge effects were determined at the systems level, with ROI-level edges included as a random effect:

$$\text{normalized weight}\sim \beta_{1}\text{group}+\beta_{2}sex+\beta_{3}age+\beta_{4}TBV+\left(1|ROI\;\text{edge}\right)$$ (4B)

The effect size associated with the group term was estimated as the actual case–control effect size for a given region or edge. Then, for each region and edge, 1,000 permutations were run in which the group label was randomly sampled, and the case–control effect size was estimated under the null hypothesis. The *Z* score for each actual effect size in this permutation distribution was calculated; any region with absolute value *Z* score > 1.96 (*p* = 0.05) was considered significant.

#### Relating developmental changes and case–control stress effects.

To characterize the relationship between RMS case–control effects and normative developmental changes, Pearson’s correlation was run on Δ*w*_dev_ and Δ*w*_age_ versus edge-level PND 63 case–control effect size. To assess the extent to which the strength of this relationship was greater than expected under the null hypothesis, 10,000 permutations were run, in which the edge assignment of RMS effect size was randomly resampled and again correlated with Δ*w*_dev_ and Δ*w*_age_. The *Z* score of the actual Pearson’s *r* was determined, and the *p* value was calculated as 1 − (proportion of permuted correlations that were smaller than the observed correlation).

For each analysis, *p*-values were corrected for multiple comparisons using the Benjamini-Hochberg method to control the false discovery rate (FDR).

### Rat Brain Plot Construction

#### Flatmap rendering.

A flatmap is a two-dimensional representation of the brain that facilitates visualization of spatial relationships between regions (Herrick, 1948; Swanson, 2000). Flatmaps are particularly useful for displaying cortical organization and topographic continuity, avoiding distortions introduced by sulcal and gyral folding in three-dimensional renderings (Swanson, 2000). While flatmapping techniques have been used elsewhere in comparative neuroanatomy and cortical cartography (Herrick, 1948; Nauta & Karten, 1970), Swanson and colleagues were the first to systematically adapt this approach to the rat brain (Hahn & Duckworth, 2023; Hahn et al., 2021; Swanson, 1992). In this study, rat brain flatmap plots were derived from the work of Hahn and Duckworth (2023) and Hahn et al. (2021).

The scalable vector graphics (SVG) file provided by Hahn and Duckworth (2023) was first converted to a well-known text (WKT) using a code from https://github.com/davidmcclure/svg-to-wkt. The WKT was then parsed into geometry objects in R using the package sf version 1.0.17 (Pebesma, 2018). Flatmaps represent a practical and underutilized tool for visualizing architectonic variation and similarity in the rat cortex, particularly in connectomic studies. To facilitate broader use, we provide all flatmap construction resources.

#### Anatomical rendering.

Rat brain maps were also visualized using an anatomical rendering that preserved three-dimensional brain structure. Nifti files representing the right and left hemispheres of the WHS (generated in edge distance calculation) were loaded into R using the package RNifti version 1.7.0 (Clayden et al., 2024). Each region was converted to a point cloud based on voxel intensity index using the R package lidR version 4.1.2 (Roussel et al., 2020). Outlying voxels (noise) were removed using the isolated voxel filter (resolution = 4, *N* = 25; Roussel et al., 2020). Point clouds were converted to geometry objects in R using the package sf version 1.0.17 (Pebesma, 2018).

## RESULTS

### Defining and Describing the Normative Network

**Figure 2.**
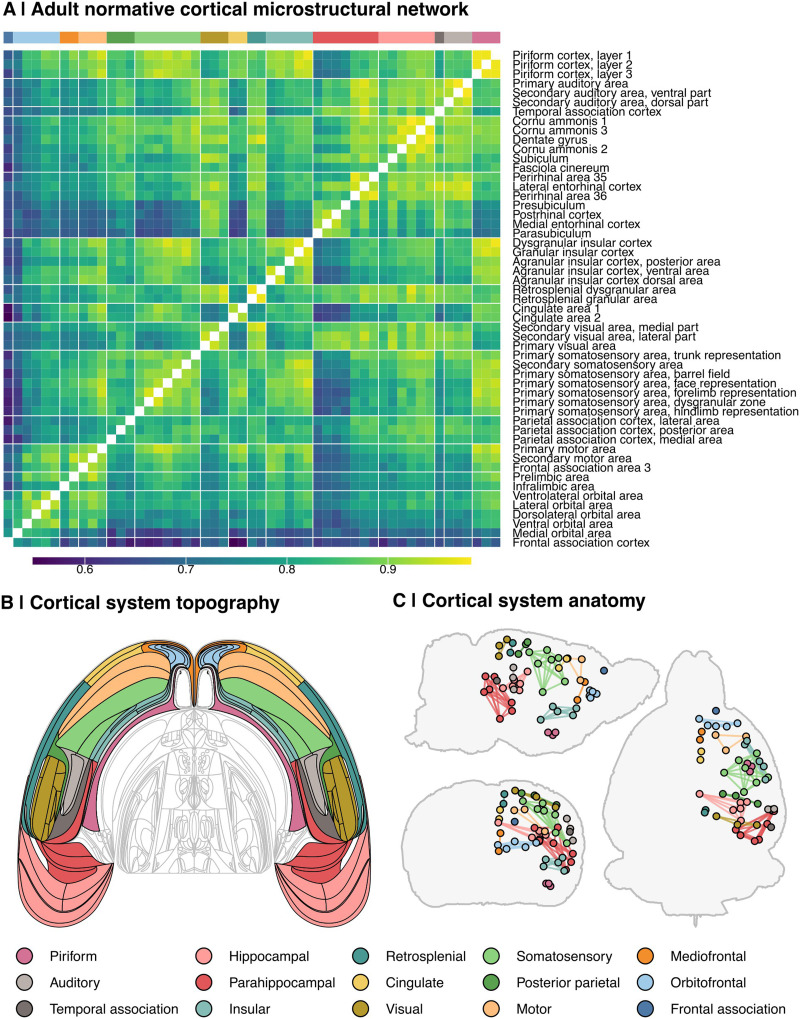
Structure of the normative rat cortical microstructural network. (A) Heatmap representation of the normative rat connectome, defined as the median edge weight across rats in the normative cohort, at PND 63. Rows and columns are ordered by cortical systems, as indicated by annotation bars. Tile color indicates strength of MIND similarity (edge weight, *w*). (B) A flatmap rendering of cortical systems derived from Hahn et al. (2021) and Hahn and Duckworth (2023). Colors correspond to cortical systems as indicated below. (C) Anatomical positions of regions of interest within each cortical system. Each point represents the center for a given cortical region of interest (*N* = 53), as defined by the WHS; lines represent intrasystem edges. For coronal and axial planes, only right-hemisphere nodes are shown.

We calculated each individual rat cortical network as the {53 × 53} matrix of edge weights (*w*), representing all pairwise MIND similarity values between 53 brain regions. To estimate the normative cortical microstructural network, we computed the median of each edge weight across the 41 male adult rat brains (PND 63; young adulthood) from the normative cohort (Figure 1A, Figure 2A, Table 1, Supporting Information Table S3). The 53 regions were grouped into 15 coarse-grained cortical systems, as defined by the WHS atlas (Kleven et al., 2023), for edge-level analyses and interpretability of results (Figures 1B and 1C).

### Normative Network Topology

**Figure 3.**
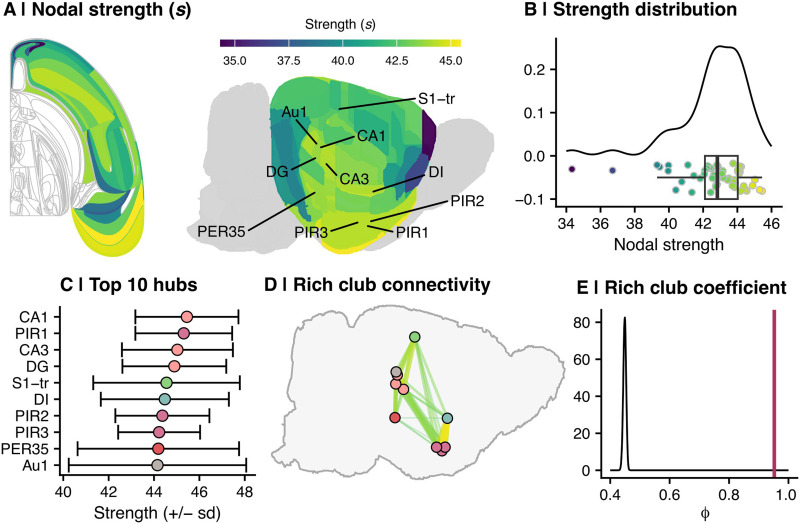
Graph-based metrics of the normative network. (A) Maps of normative nodal strength. Left: Topographic (flatmap) cortical rendering (right hemisphere only). Right: Anatomical MRI rendering. Network hubs, or top 10 nodes by strength, are labeled. CA1 = cornu ammonis 1; PIR1 = piriform cortex, layer 1; CA3 = cornu ammonis 3; DG = dentate gyrus; S1-tr = primary somatosensory area, trunk representation; DI = dysgranular insular cortex; PIR2 = piriform cortex, layer 2; PIR3 = piriform cortex, layer 3; PER35 = perirhinal area 35; Au1 = primary auditory area. (B) Nodal strength distribution of the adult normative network. Each point on the *x*-axis shows the strength of a given region (defined as the sum of all edge weights for that region), while the *y*-axis shows smoothed density of nodes for each strength bin. (C) Ten regions with the highest nodal strength, that is, “hubs.” The *x*-axis represents the median strength (± standard deviation) of each region; each point is colored by cortical system as in Figure 2. The *y*-axis shows each region of interest (same abbreviations as panel A). (D) Anatomical representation of the network rich club. Each point represents a hub (from panel C), and the interconnectivity among all hubs is indicated by connecting lines. The width and color of each line indicates edge weight, that is, the strength of similarity between the two hubs. (E) Normative MIND network rich club coefficient versus 10,000 distance-corrected null networks. The *x*-axis shows the rich club coefficient, while the *y*-axis indicates density distribution. The rich club coefficient of the normative network, defined as the sum of all hub interconnections (*N* = 90) divided by the sum of the top *N* = 90 edges, is shown in by the position of the maroon line (*Φ* = 0.95). The black curve shows the distribution of rich club coefficients across distance-corrected null networks (median *Φ* = 0.45).

The normative network distributions of nodal strength (i.e., the sum of all edge weights connecting it to the rest of the network, *s*; Figure 3A) and edge weights were left-skewed (Figure 3B), indicating a generally high MTR-based anatomical similarity across the cortex. Hubs, defined as regions with the highest strength (Fornito et al., 2016b), and rich-club organization, which examines whether hubs have stronger similarity with each other than expected by chance (Fornito et al., 2016a), are functionally relevant features of brain networks described in humans and other organisms (Bullmore & Sporns, 2009; Rubinov et al., 2015; Swanson et al., 2024b; Towlson et al., 2013). We found this principle held true in rat brain MIND networks. The hubs, defined as the 10 nodes with the highest strength, were largely hippocampal and piriform regions (Figure 3C). The rich club coefficient (*Φ*), calculated using Equation 1, was 0.95, compared with a median coefficient of 0.45 across 10,000 distance-corrected null networks (Figures 3D and 3E), indicating a significantly greater-than-random strength of connectivity among cortical hubs (*Z* = 73; *P*_perm_ = 1.0 × 10^−4^). This evidence for a rich-club organization in MIND networks was consistent with prior reports of rich clubs in the meta-analytic connectome from rat tract-tracing data (Swanson et al., 2024b) and in other species and modalities of brain networks (Towlson et al., 2013; van den Heuvel & Sporns, 2011).

### Network Validation and Reliability

The neurobiological validity of MIND networks rests on two key assumptions: (a) that MRI-derived similarity at macros scale reflects cyto- and myeloarchitectonic similarity at micro scale and (b) that architectonic similarity between cortical areas serves as a proxy for their axonal connectivity. Consistent with these assumptions, research in humans and nonhuman primates has shown that structural MRI similarity networks reflect key organizational features of the cortex including patterns of cytoarchitectonic similarity (the architectome) and axonal connectivity (the connectome; Sebenius et al., 2023, 2025; Seidlitz et al., 2018). Since this study is the first to describe structural similarity networks in rats, we aimed to establish these relationships within the normative network reported here. Confirming the neurobiological relevance of this network enhanced the interpretability and significance of downstream findings.

We validated the normative MIND network against four key predictions: that MIND similarity was greater between regions that (a) were spatially closer to each other, (b) were more architectonically similar to each other, (c) had more similar gene expression profiles, and (d) had more similar profiles of axonal projections in prior tract-tracing data. All four of these predictions were verified as detailed below.

#### MIND similarity–distance relationship.

**Figure 4.**
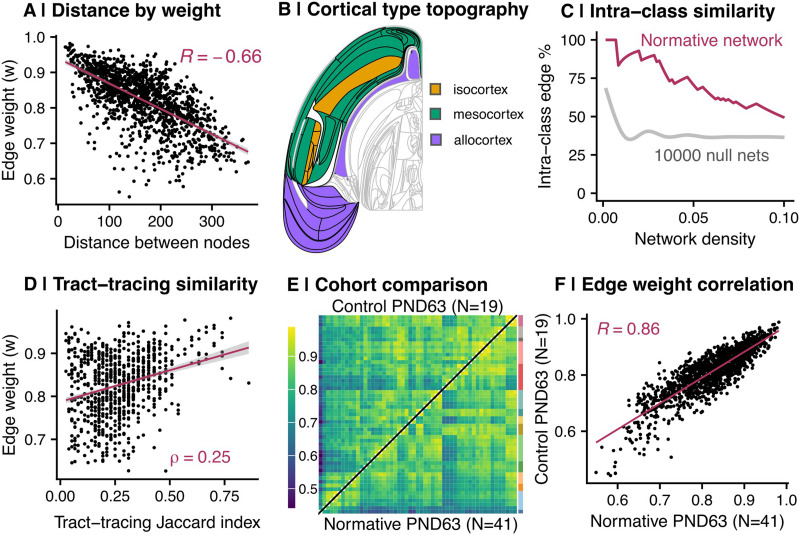
MIND-based similarity is related to metrics of cortical organization. (A) Scatter plot of the distance between two nodes (*x*-axis) and their MIND similarity, or edge-weight between them (*y*-axis). Distance is defined as the Euclidean distance between region of interest centers, as determined using the WHS atlas. (B) Topographic (flatmap) rendering of cortical types (left hemisphere only). Regions are colored by cortex type, as defined by García-Cabezas et al. (2023). (C) Proportion of intraclass edges across network density thresholds. The *x*-axis shows the proportion of top-weighted edges considered, while the *y*-axis indicates the percentage of those top-weighted edges that consists of two regions from the same cortex class. The maroon curve shows the percentage of within-class edges in the normative MIND network, while the gray line shows the mean percentage (± standard deviation) of within-class edges across 10,000 permuted null networks. (D) Correlation between similarity of tract-tracing connection profiles between pairwise combinations of regions and strength of MIND similarity. The Jaccard index for edge *ij* was defined as the intersection of tract-tracing connections (i.e., where *ik* = *jk* for all regions *k*) divided by the union of all connections containing *i, j*, or both (Equation 2). (E) Heatmap representations of the median PND 63 control network from the experimental stress cohort (top left of the diagonal) and the median PND 63 network from the normative developmental cohort (bottom right of the diagonal). Panel legend is the same as Figure 2A. (F) The relationship between edge weights in the median PND 63 network from the normative development cohort (*x*-axis) and median PND 63 control network from the experimental stress cohort (*y*-axis; *r* = 0.86; *p* < 0.001). Each point represents an edge; the line of best fit is shown in maroon.

First, we found that morphometric similarity decreased with increasing distance between regions such that the Euclidean distance between node centers was negatively correlated with edge weight (i.e., MIND similarity; *r* = −0.66; *p* < 0.001; Figure 4A). This result aligned with expectations, as brain regions that are in closer spatial proximity tend to exhibit greater structural similarity (Alexander-Bloch et al., 2013).

#### Cytoarchitectonic relationships to MIND similarity.

Second, if MIND similarity truly reflects cytoarchitectonic similarity, we would expect regions of the same cortical type to exhibit higher MIND edge weights. The rat neocortex is structured along gradients of laminar differentiation, which expanded from allocortical areas (archicortical [hippocampal] and paleocortical [olfactory]) according to the dual origin theory (García-Cabezas et al., 2023; Figure 4B). In rodents, these cortical gradients extend through mesocortical (agranular and dysgranular) into isocortical (eulaminate) areas (García-Cabezas et al., 2023). To test the relationship between MIND similarity and cytoarchitectonics, we coregistered our neuroimaging data with a cytoarchitectonic atlas of the rat brain (García-Cabezas et al., 2023; Figure 4B) and compared MIND edge weights between areas of the same cortex class (“intraclass” edges) versus areas of different cortex classes (“interclass” edges), removing isocortical regions due to small sample size (*N* = 5 regions).

Intraclass edges between two allocortical areas (“allo-allo”) had significantly higher MIND similarity than edges between allocortical and mesocortical (“allo-meso”) areas (FDR < 0.05; (Supporting Information Figure S3). However, meso intraclass edges were not significantly more similar than allo-meso. To probe this relationship further, we calculated the percentage of intraclass edges (allo-allo and meso-meso) at various binary network densities, thresholding across a range of sparse connection densities (1%–10% of all possible pairwise edges). We then compared these with distance-corrected null networks thresholded across the same range (Figure 4C). Across all thresholds, the empirical rat network demonstrated a higher percentage of intraclass edges than the null networks (Figure 4C), indicating a higher-than-chance density of cytoarchitectonically similar edges among edges with the highest MIND similarity.

#### Mouse transcriptional relationships to MIND similarity.

Third, we predicted that regions with more similar patterns of gene expression would also exhibit higher MIND similarity. However, a spatial transcriptomic atlas of the rat brain is not yet available. To overcome this limitation, we leveraged homology between the rat and mouse brain to access spatially comprehensive transcriptomic data from the AMBA (*N* = 437 genes; Yao et al., 2023). While we acknowledge the challenges of cross-species comparisons, our goal was to assess the general consistency between edge-wise MIND similarity and transcriptional similarity, rather than expecting a precise one-to-one correspondence.

We tested and verified that regions with higher MIND similarity also demonstrated higher transcriptomic similarity, calculated as the pairwise regional correlation between gene expression profiles (Supporting Information Figure S4). These findings support our general hypothesis that MIND similarity reflects broad patterns of transcriptomic similarity.

#### Tract-tracing relationships to MIND similarity.

Fourth, we hypothesized that MIND similarity would reflect patterns of axonal connectivity. To test this, we converted meta-analytic rat tract-tracing data (Swanson et al., 2024b) to a similarity matrix by calculating the Jaccard index of the log_10_-transformed ordinal connection weight between pairwise regions (Equation 2; Supporting Information Figure S5A). This transformation yielded a continuous measure representing the similarity of axonal connectivity profiles between regions (i.e., the extent to which two regions share common connectivity patterns).

When the tract-tracing Jaccard index was correlated with MIND edge weights, we found a positive relationship (*ρ* = 0.25; *p* < 0.001; Figure 4D). To assess the significance of this correlation, we compared it with a null distribution generated from 10,000 distance-corrected null networks. The observed Spearman correlation was significantly greater than chance (*Z* = 6.04; *P*_perm_ = 1.0 × 10^−4^; Supporting Information Figure S5B), suggesting a meaningful relationship between anatomical connectivity profiles and MIND similarity.

#### Cross-cohort reproducibility.

Finally, to assess the reliability of MIND network analysis, we directly compared the median adult MIND network for the normative developmental cohort (*N* = 41, PND 63; Figure 1A) to the median adult MIND network for an independent cohort of rats: namely, the normally reared (control) group in the experimental stress cohort (*N* = 19, PND 63; Figure 1B and Figure 4E). Edge weights were highly correlated between the normative adult and independent control MIND networks (*r* = 0.86; *p* < 0.001; Figure 4F), as was nodal strength (*r* = 0.91; *p* < 0.001). These results demonstrate a high level of replicability of the cortical microstructural network estimated by identically implemented MIND analyses of MTR data collected using the same sequences in two independent cohorts of rats.

### Normative Developmental Changes in the Rat Cortical Microstructural Network

**Figure 5.**
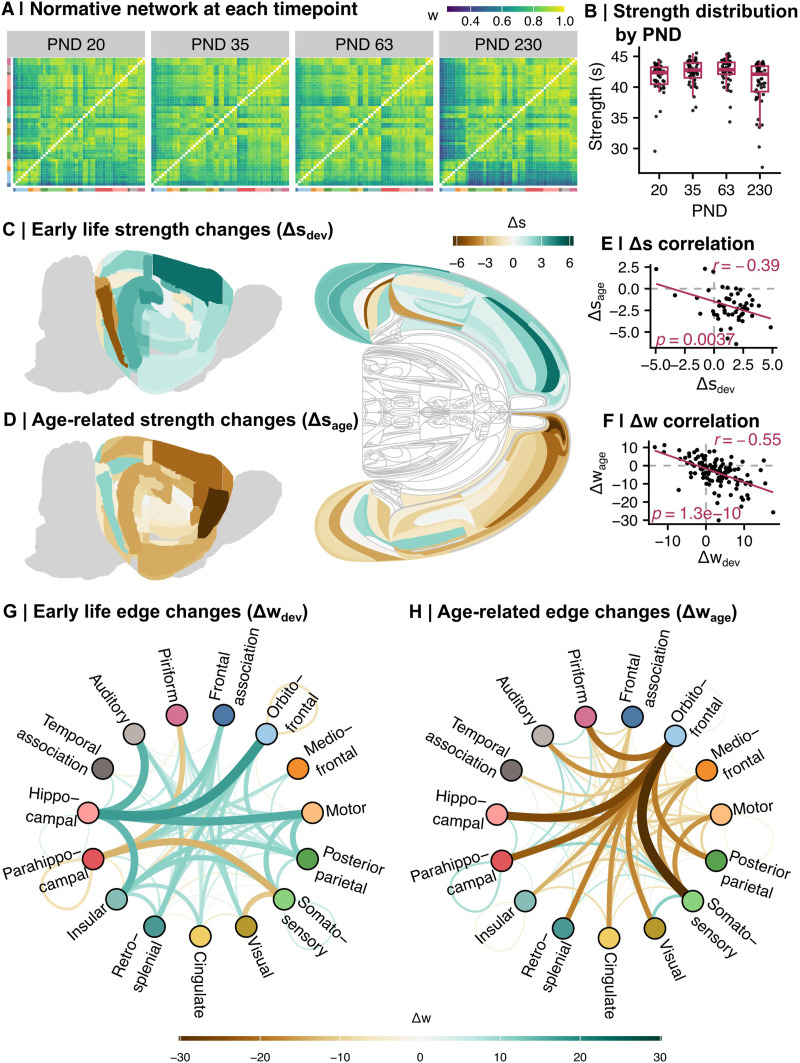
The normative rat cortical connectome generally increases in similarity in early development and decreases in similarity in aging. (A) Heatmap representation of the connectome throughout development in the normative cohort (median edge weight, at each timepoint). Rows and columns are ordered by decreasing-to-increasing nodal strength within broader cortical system (in the same order as in Figure 2A). Tile color indicates strength of MIND similarity (edge weight, *w*). (B) The normative strength distribution through development, defined as the median strength per ROI at each timepoint. Each point represents a region of interest; boxplots show the overall strength (*s*) distribution at each timepoint. The normative strength distribution at PND 20 is significantly lower than the distribution at PND 63 (Dunn test *p* = 0.019); the nodal strength distribution at PND 230 is significantly lower than the distributions at PND 35 (Dunn test *p* = 0.027) and PND 63 (Dunn test *p* = 0.003; Kruskal–Wallis *p* = 0.002). (C) Anatomical patterning of nodal strength changes in early development (Δ*s*_dev_). Left: Volumetric rendering; right: flatmap rendering (left hemisphere/top half only; Hahn & Duckworth, 2023; Hahn et al., 2021). Brown indicates decrease in strength, teal indicates increase in strength. Δ*s*_dev_ was considered significant if |*t*-value| of the age term in the mixed effects model was greater than 2. (D) Anatomical patterning of nodal strength changes in aging (Δs_age_; flatmap rendering right hemisphere/bottom half). Figure key the same as panel C. (E) Pearson correlation between Δs_dev_ (*x*-axis) and Δs_age_ (*y*-axis; *r* = −0.39; *p* = 0.004). Each point represents a region of interest; the line of best fit is shown in maroon. (F) Pearson correlation between Δw_dev_ (*x*-axis) and Δw_age_ (*y*-axis; *r* = −0.55; *p* < 0.001). Each point represents an edge; the line of best fit is shown in maroon. (G) A circle plot of significantly changed system-level edges in early development (Δ*w*_dev_; |*t*| > 3.3). Each datapoint in the circle represents a broader cortical system, colored and labeled by system. The color and size of the curves connecting two points show the change in edge weight (brown indicates decreasing similarity; teal indicates increasing similarity). (H) A circle plot of significantly changed edges in aging (Δ*w*_age_). Figure key the same as panel G.

Having established convergent validity and intersample replicability of MIND networks, we next harnessed this analytic approach to model network-level reorganization of the rat brain over development. This strategy, applied to a longitudinal MRI dataset spanning PND 20, 35, 63, and 230 (*N* = 162 total scans, Figure 1A, see the Methods section, Table 1), revealed developmental changes in (a) each region’s morphometric similarity with the rest of the brain (strength, *s*) and (b) the morphometric similarity (edge strength, *w*) between each unique pair of regions. Visual inspection of the median MIND matrices for each of the four time-points indicated that there are age-related changes in the cortical pattern of interareal similarity. For example, frontal cortical areas (frontal association cortex, orbitofrontal cortex, mediofrontal cortex, and motor areas) become more similar to the rest of the cortex during early development and then strikingly less similar during later aging (Figure 5A). Likewise, nodal strength showed a general tendency to increase during development and decrease during aging (Figure 5B).

We quantified the regional rate of change in early development as the linear gradient or slope of age-related change in nodal strength for each cortical area between PND 20 (weanling) to PND 35 (adolescence), Δ*s*_dev_, accounting for intersubject variation (Equation 3A). Similarly, to calculate changes in aging, we applied the same model to estimate the age-related change in strength for each cortical area from PND 63 (young adult) to PND 230 (mid adult) timepoints, Δ*s*_age_.

During development, regions in most cortical systems significantly increased in nodal strength, especially the hippocampal formation and motor cortex (Δ*s*_dev_ *t* values = 7.19 and 5.22, respectively; Figure 5C). The parahippocampal region decreased in similarity with the rest of the brain (Δ*s*_dev_ *t* = −1.34). In contrast to these developmental changes, most cortical systems decreased in nodal strength during aging, especially areas of the frontal cortex, including the orbitofrontal (Δ*s*_age_ *t* = −11.4), motor (Δ*s*_age_ *t* = −6.13), and mediofrontal (Δ*s*_age_ *t* = −5.94; Figure 5D) cortices. Early life and aging effects on nodal strength were negatively correlated (*r* = −0.39; *p* = 0.004; Figure 5E), indicating that those regions showing the strongest strength increases in development also tended to show the most rapid strength decreases in aging. Ten fronto-hippocampal regions (frontal: dorsolateral orbital area, frontal association cortex, frontal association area 3, primary motor area, secondary motor area; hippocampal: dentate gyrus, perirhinal area 35, cornu ammonis 1) both significantly increased in strength in early development and significantly decreased in strength in aging. Only one region, the parasubiculum, significantly decreased in early development and increased in aging.

We also calculated changes in early development and aging for each interareal similarity or edge in the MIND networks (Δ*w*_dev_ and Δ*w*_age_, respectively; Equation 3B). In early development, the hippocampal formation demonstrated notable increases in similarity with frontal regions, including the orbitofrontal (Δ*w*_dev_ *t* = 17.6), motor (Δ*w*_dev_ *t* = 15.5), frontal association (Δ*w*_dev_ *t* = 11.7), and mediofrontal (Δ*w*_dev_ *t* = 7.97) cortices (Figure 5G). In contrast, parahippocampal regions largely decreased in similarity with other cortical systems, including the somatosensory (Δ*w*_dev_ *t* = −13.4) and piriform (Δ*w*_dev_ *t* = −11.0) cortices. In aging, the most salient changes in edge weight involved orbitofrontal regions, which significantly diverged from every other cortical system, most notably the somatosensory (Δ*w*_age_ *t* = −30.1), hippocampal (Δ*w*_age_ *t* = 25.7), and parahippocampal (Δ*w*_age_ *t* = 22.5) regions (Figure 5H). As with strength, edge weight effects during development and aging were negatively correlated (*r* = −0.55; *p* < 0.001; Figure 5F), demonstrating that frontal and hippocampal systems with the most rapid increases in pairwise similarity during early development showed the fastest decreases in similarity, or increases in dissimilarity, during aging.

### Early Life Environmental Stressors Perturb Adult Cortical Similarity Networks

**Figure 6.**
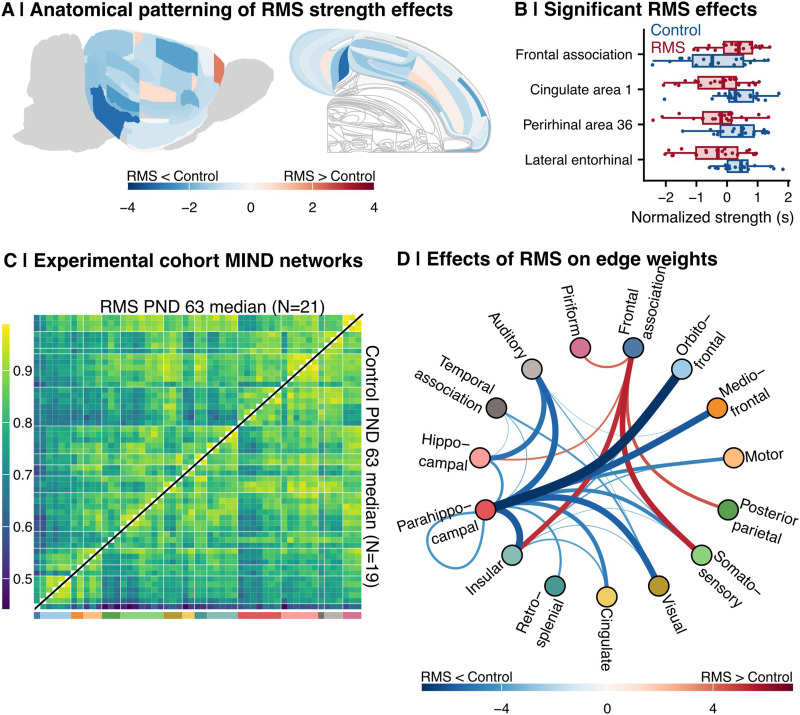
Impact of early life stress on nodal strength and edge weights. (A) Anatomical distribution of the post-natal day 63 (young adulthood) RMS-control effect sizes across brain slices. For each region of interest, a linear model was run of the normalized strength on group + age + sex + TBV, and the group statistic was extracted as the actual effect size. Then, 1,000 permutations were run in which the group assignments were shuffled, the same linear model was run, and the group statistic was extracted as the permuted effect size. The *Z* score of the actual effect size was calculated as its position in the permuted distribution (see the Methods section). Regions with positive *Z* scores (shown in red) demonstrated increased strength in RMS subjects in young adulthood; regions with negative *Z* scores (shown in blue) demonstrated decreased strength. The flatmap rendering was derived from Hahn and Duckworth (2023) and Hahn et al. (2021). (B) Boxplots showing the nodal strength distribution by group for the four regions with significant case–control differences at PND 63 (|*Z*_perm_| > 1.96). The *x*-axis shows nodal strength, corrected for covariates and normalized for visualization purposes. Each point represents a subject, while the box-and-whiskers plot shows the overall distribution by group. Blue indicates control; red indicates RMS. (C) The median PND 63 MIND networks in control subjects (bottom right) versus RMS subjects (top left). The figure legend is the same as in Figure 2A. (D) A circle plot of significant RMS-control edge weight differences in young adulthood (PND 63; |*t*| > 3.3). Node order and system annotation are the same as in Figure 5 panels G and H. Curves connecting two points indicate a significant case–control difference in edge weight; colored by effect size (with red indicating higher weight in RMS and blue indicating higher weight in control); line width indicates absolute value effect size.

We assessed the impact of early life stress—as modeled by RMS—on the nodal strength of the young adult cortical microstructural network by running case–control comparisons for each cortical area at the PND 63 timepoint (Equation 4A; Figure 1B). Exposure to RMS was associated with strength decreases in most brain regions (*N* = 40 of 53 total; Figure 6A). Four regions had significant differences following permutation testing: The lateral entorhinal cortex, perirhinal area 36, and cingulate area 1 decreased in strength in RMS (permutation *Z* scores = −3.07, −2.31, and −2.18, respectively), while the frontal association cortex demonstrated increased strength following RMS (*Z*_perm_ = 2.08; Figure 6B).

Qualitatively, visual inspection of the median MIND matrices in control (*N* = 19) and RMS-exposed rats (*N* = 21) indicated that the early life stressor induced changes in the cortical pattern of interareal similarity, especially in frontal and hippocampal systems (Figure 6C). Indeed, edge-level case–control analyses (Equation 4B) showed that RMS-exposed rats had strongly decreased similarity between parahippocampal cortex and several other cortical areas (including the orbitofrontal cortex (*t* = −6.89), mediofrontal cortex (*t* = −5.89), and motor cortex (*t* = −4.69; Figure 6D). This contrasts with markedly increased similarity between the frontal association cortex and other areas, including the somatosensory (*t* = 5.40) and insular cortices (*t* = 5.17). Together, these results indicate vulnerability in network structure to environmental stress, especially in frontal and parahippocampal regions most sensitive to developmental and aging changes in nodal strength.

### Effects of Early Life Stress Are Nested Within Normative Developmental Changes in the Cortical Microstructural Network

Finally, we tested more formally for potential congruence between stress effects and normative network changes in development and aging. Strikingly, variation in the effects of RMS on interareal edge weights in the rat cortical microstructural network was positively correlated with variation in the edge weight changes over normative development (Δw_dev_; *r* = 0.18, *P*_perm_ = 0.03; *Z*_perm_ = 1.90; Supporting Information Figure S6 [left]). Specifically, those edges showing greatest similarity increases in normative development also tended to show greater similarity increases following RMS. Furthermore, the effects of RMS on edge weight were significantly and negatively correlated with normative age-related changes in edge weight (Δw_dev_; *r* = −0.19, *P*_perm_ = 0.02; *Z*_perm_ = −2.03; Supporting Information Figure S6 [right]), indicating that the edges with the greatest similarity increases following RMS were also those that decreased in similarity the most in normative aging. Taken together, these results are consistent with a model in which early life stress accelerates normative brain reorganization during development, and areas which are most developmentally dynamic and vulnerable to stress are also the most susceptible in aging.

## DISCUSSION

We have pioneered and validated MIND similarity network analysis as a novel approach to inferring myelo-architectonic similarity between all cortical areas in an individual rat’s brain. Using this methodological advance, we studied *N* = 47 rat cortical networks to investigate normative dynamics in cortical similarity across development and aging. These changes, which primarily involved frontal and hippocampal systems, were examined through MIND analysis of myelin-sensitive MTR data collected up to four times over each rat’s lifespan. In a second experiment, we tested the hypothesis that early life stress exposure was associated with subsequent abnormalities of cortical network organization. RMS in the first 20 days of postnatal life affected the adult similarity of cortical areas, especially frontal and hippocampal systems that are normatively most dynamic in adolescent development and early brain aging.

Structural MRI similarity analysis is being increasingly used as a measure of brain network organization in various methodological and experimental contexts (Sebenius et al., 2025, and references therein). In general, the interpretation of MIND similarity rests on two key assumptions: (a) that the correlation or inverse divergence between MRI features in two cortical areas reflects their cyto-architectonic or myelo-architectonic similarity and (b) that cortical areas that are more architectonically similar are more likely to be axonally interconnected (Bazinet et al., 2023; Sebenius et al., 2025). We can therefore think of a structural MRI similarity network as primarily a map of cortical patterning—an architectome—which is in turn a partial proxy for the map of axonal wiring—a connectome. To validate rat brain MIND similarity analysis, we tested both these key assumptions. The results confirmed that cortical areas belonging to the same architectonic class, or spatially adjacent to each other, tended to have higher MIND similarity, and that higher MIND similarity between cortical areas was associated with stronger evidence of similarity in axonal connectivity, based on a prior meta-analysis of tract-tracing studies (Swanson et al., 2024b). Since the MRI data we used for this analysis were collected using an MT (MTR) sequence that is known to be sensitive to cortical myelination and neuropil density (Grossman et al., 1994), we can therefore generally interpret MTR-derived MIND (dis-)similarity as indicative of architectonic differentiation and myelination of the cortex of an individual rat.

To characterize the dynamics of such differentiation across the lifespan, we measured changes in similarity in early life, defined as PND 20 to 35, and in later life, defined as PND 63 to 230. Though the data on developmental patterns of rat brain myelination are sparse, a histological study demonstrated that the rat brain begins myelinating around PND 10, and most areas are fully myelinated by PND 24 (Downes & Mullins, 2014). Thus, our “early” developmental data likely reflect the end of early life myelinating processes (e.g., late adolescence) and do not capture earlier peaks in cortical myelination. We show that increasing MIND similarity in fronto-hippocampal circuitry in late adolescent development (“early life”) is coupled to rapidly decreasing similarity of these systems in mid-adulthood aging. These data thus support the “last in, first out” hypothesis of development and aging, in which plastic regions that mature last in early development (namely, frontal areas related to decision-making and executive function) are more vulnerable to age-related decline, as previously described in humans (Douaud et al., 2014; Duan et al., 2024; Fjell et al., 2014). We demonstrate here that rat brain microarchitecture is subject to the same phenomenon.

Developmentally sensitive fronto-(para)hippocampal circuitry also showed targeted disruptions in young adult rats who had been exposed to RMS. This finding is consistent with extensive prior work showing that maternal separation alters the structure and function of fronto-limbic circuits, including morphological and functional changes in the prefrontal cortex (particularly orbitofrontal subregions), cingulate cortex, and hippocampus (for comprehensive reviews, see Cohodes et al., 2021; Speranza et al., 2024). Notably, however, analyses of these same data using conventional regional volumetrics revealed less pronounced RMS effects than the MIND approach (Dutcher et al., 2023), highlighting methodological differences that may complicate direct comparisons. Our result also aligns with studies in humans, in which later-developing plastic circuitry also shows increased vulnerability to disruptions during early development and aging, such as schizophrenia and Alzheimer’s disease, respectively (Douaud et al., 2014; Fjell et al., 2014). The congruence between developmentally dynamic and environmentally sensitive regions was reinforced quantitatively, as edges that increased in similarity in development tended to show higher similarity following RMS (Supporting Information Figure S6). These results are consistent with an accelerated development hypothesis of early life stress (Callaghan & Tottenham, 2016). Edges that showed higher similarity in young adulthood following early life stress also demonstrated more rapid divergence in normative aging. This provides further support for the concept that specific fronto-hippocampal circuits are developmentally dynamic, vulnerable to age-related decline, and susceptible to environmental stress (Douaud et al., 2014; Duan et al., 2024). The alignment between human and rat data underscores the potential of this model system for experimental investigation into the mechanisms linking early life stress, network development, and aging.

An increase in similarity between two areas (in this case, frontal and hippocampal) does not necessarily indicate the formation of new axonal connections but likely represents coordinated changes in (a) myelination of fibers and/or (b) microstructural properties, such as synaptic or dendritic remodeling. The first hypothesis is supported by rat histological data showing that, between PND 24 and PND 37, myelination occurs exclusively in the fornix and mammillothalamic tract—pathways that traverse the hippocampus (Downes & Mullins, 2014). It has been argued theoretically that regions related to memory and learning develop as the adolescent rat ventures out and requires spatial recognition, a skill less essential earlier in life (Downes & Mullins, 2014). Consistent with this, a study on rat hippocampal myelination reported the first appearance of myelinated fibers at PND 17, a significant increase to near-adult levels by PND 25, and full maturation by PND 60 (Meier et al., 2004).

In addition to myelination, changes in MIND similarity may also arise from coordinated microstructural reorganization. Extensive cross-modality research highlights hippocampal plasticity in rodents, especially changes in synaptic density in response to environmental enrichment or deprivation (Bramati et al., 2023; Dayananda et al., 2023; Lerch et al., 2011; Ohline & Abraham, 2019; Stein et al., 2016). Similarly, the frontal cortex undergoes environmentally sensitive synaptogenesis in early development—continuing into adulthood in rats (Zeiss, 2021)—and synaptic pruning throughout the lifespan (Kolk & Rakic, 2022). These shifting neuropil profiles suggest that cortical regions without direct physical connections may exhibit high MIND similarity due to convergent synaptic architectures—or increasingly divergent profiles with other regions. Our cross-validation data support this concept. In the tract-tracing (Swanson et al., 2024b) Jaccard comparison, a subset of edges showed low tract-tracing similarity but high MIND similarity, predominantly involving hippocampal regions. Excluding hippocampal edges strengthened the correlation between MIND similarity and tract-tracing (*ρ* = 0.41; *p* < 0.001; Supporting Information Figure S5C). We postulate that the observed high MIND similarity, despite low axonal connection similarity, may reflect convergent plastic reorganization of the microstructural properties of these regions. Notably, the hypotheses of cortical fiber myelination and synaptic density as contributors to MIND similarity are likely interconnected, as evidence suggests neural activity can induce myelinogenesis (Demerens et al., 1996). Future studies incorporating histological measures of synaptic density and activity-dependent myelination in the rat brain could help clarify the relative contributions of these mechanisms to MIND similarity.

A key limitation of this study is that the normative developmental cohort included only male animals; accordingly, the normative network defined here represents male development only. To more fully characterize normative development, future work should explicitly investigate sex differences in network structure and dynamics. It would also be of interest to reapply these methods using varying parcellation schemes, as different definitions of brain regions—both cortical and subcortical—may offer complementary or more detailed insights. Future work to characterize MRI-derived rat brain network architecture could include additional morphometric features, such as diffusion tensor imaging metrics, within a multivariate MIND analysis. This may provide a broader view of cortex-wide morphological covariation and more directly reflect axonal connections. As additional rat brain resources become available (for instance, a brain-wide spatial transcriptomic atlas and consensus nomenclature and parcellations), our ability to biologically annotate the rat structural similarity network will continue to improve.

We provide the normative MTR-derived rat cortical microstructural network and all associated code to support further investigation and understanding of the complex organization of cortical networks in this key model system. Our results demonstrate the biological validity and replicability of the MIND similarity analysis and demonstrate its sensitivity to developmental and environmental stress-related changes in cortical network configuration. We emphasize the importance and vulnerability of key frontal and hippocampal circuitry in dynamic processes of normative development and atypical developmental trajectories triggered by early life adversity.

## Acknowledgments

At the time of this work, R.L.S. was a PhD candidate in the National Institutes of Health (NIH) Oxford-Cambridge Scholars Program. R.L.S., P.A.T., D.R.G., A.R., and F.J.M. National Institute of Mental Health (NIMH) Intramural Research Program of the NIH/HHS, USA as follows: R.L.S. and F.J.M. (1ZIAMH002810), A.R. (1ZIAMH002949), P.A.T. and D.R.G. (ZICMH002888). L.D. and E.G.D. were supported by the Gates Cambridge Scholarship. P.E.V. was supported by MQ: Transforming Mental Health (MQF17_24). E.T.B. was also supported by an National Institute for Health and Care Research (NIHR) Senior Investigator award. This work received computational support from the NIH High Performance Computing (HPC) Biowulf cluster (https://hpc.nih.gov) and the NIMH Scientific and Statistical Computing (AFNI) Core. This research was supported [in part] by the Intramural Research Program of the NIH. The contributions of the NIH author(s) are considered Works of the United States Government. The findings and conclusions presented in this paper are those of the author(s) and do not necessarily reflect the views of the NIH or the U.S. Department of Health and Human Services. All research at the Department of Psychiatry in the University of Cambridge is supported by the NIHR Cambridge Biomedical Research Centre (NIHR203312) and the NIHR Applied Research Collaboration East of England. The views expressed are those of the author(s) and not necessarily those of the NIHR or the Department of Health and Social Care.

## Supporting Information

Supporting information for this article is available at https://doi.org/10.1162/NETN.a.546.

## Author Contributions

Rachel L. Smith: Conceptualization; Data curation; Formal analysis; Investigation; Methodology; Software; Validation; Visualization; Writing – original draft. Stephen J. Sawiak: Data curation; Funding acquisition; Investigation; Methodology; Resources; Software; Writing – review & editing. Lena Dorfschmidt: Methodology; Software; Writing – review & editing. Ethan G. Dutcher: Data curation; Investigation; Methodology; Writing – review & editing. Jolyon A. Jones: Data curation; Investigation; Methodology; Writing – review & editing. Joel D. Hahn: Resources; Visualization; Writing – review & editing. Olaf Sporns: Methodology; Resources; Writing – review & editing. Larry W. Swanson: Methodology; Resources; Writing – review & editing. Paul A. Taylor: Methodology; Resources; Software; Writing – review & editing. Daniel R. Glen: Methodology; Resources; Software; Writing – review & editing. Jeffrey W. Dalley: Data curation; Funding acquisition; Methodology; Project administration; Resources; Writing – review & editing. Francis J. McMahon: Conceptualization; Supervision; Writing – original draft; Writing – review & editing. Armin Raznahan: Conceptualization; Supervision; Writing – original draft; Writing – review & editing. Petra E. Vértes: Conceptualization; Methodology; Project administration; Resources; Supervision; Writing – original draft; Writing – review & editing. Edward T. Bullmore: Conceptualization; Funding acquisition; Project administration; Resources; Supervision; Writing – original draft; Writing – review & editing.

## Competing Interests

E.T.B. has consulted for SR One, GSK, Sosei Heptares, Boehringer Ingelheim, Novartis, and Monument Therapeutics. P.E.V. has consulted for LinusBio.

## Funding Information

Jeffrey W. Dalley, GlaxoSmithKline (https://dx.doi.org/10.13039/100004330), Award ID: 300034212. Jolyon A. Jones, Medical Research Council (https://dx.doi.org/10.13039/501100000265), Award ID: PJAG/371. Petra E. Vértes, MQ: Transforming Mental Health (https://dx.doi.org/10.13039/100011705), Award ID: MQF17_24. Lena Dorfschmidt, Gates Cambridge Trust (https://dx.doi.org/10.13039/501100005370). Ethan G. Dutcher, Gates Cambridge Trust (https://dx.doi.org/10.13039/501100005370). Francis J. McMahon, National Institute of Mental Health (https://dx.doi.org/10.13039/100000025), Award ID: 1ZIAMH002810. Armin Raznahan, National Institute of Mental Health (https://dx.doi.org/10.13039/100000025), Award ID: 1ZIAMH002949. Paul A. Taylor, National Institute of Mental Health (https://dx.doi.org/10.13039/100000025), Award ID: ZICMH002888. Daniel R. Glen, National Institute of Mental Health (https://dx.doi.org/10.13039/100000025), Award ID: ZICMH002888. Edward T. Bullmore, NIHR Cambridge Biomedical Research Centre (https://dx.doi.org/10.13039/501100018956), Award ID: BRC-1215-20014. Petra E. Vértes, NIHR Cambridge Biomedical Research Centre (https://dx.doi.org/10.13039/501100018956), Award ID: BRC-1215-20014. Rachel L. Smith, NIH Oxford-Cambridge Scholars Program.

## Data and Code Availability

All data were made available through previous publications (Dutcher et al., 2023; Jones et al., 2024). All visualizations were rendered using the R package ggplot version 3.5.1 (Wickham, 2016). Code for data preprocessing (including the rat MRI preprocessing pipeline), data analysis, and figure construction is available at https://github.com/rlsmith1/rat_MRI_similarity_networks.

## Supplementary Material



## TECHNICAL TERMS

Morphometric INverse Divergence (MIND): A method that quantifies structural similarity between brain regions by calculating the Kullback–Leibler divergence between their MRI feature distributions.
Connectome: A comprehensive network or “wiring diagram” of all axonal connections (edges) between cortical areas or neurons (nodes).
Cortical similarity network: A network representation where nodes represent cortical areas and signed, weighted edges represent the structural or morphometric similarity (or dissimilarity) between areas.
Magnetization transfer ratio (MTR): An MRI measure sensitive to the density of macromolecules, frequently used as a proxy for cortical myelination.
Repeated maternal separation (RMS): A common rodent model of early life stress involving daily, timed separation of pups from their dam.
Kullback–Leibler (KL) divergence: A statistical measure used in the MIND algorithm to estimate the divergence between two univariate or multivariate distributions of MRI features.
Nodal strength: An alternative term for weighted degree, indicating the sum of all edge weights for a specific cortical node.
Rich-club organization: A network property where high-degree nodes (hubs) are more densely interconnected with each other than expected by chance.
Architectome: A map representing the spatial patterning of structurally similar (or differentiated) areas across the cortical sheet. Cytoarchitectonically similar areas of cortex are more likely to be axonally connected; hence, the architectome can be regarded as a proxy for the connectome.
“Last in, first out” hypothesis: A theory suggesting that brain regions maturing last in development (i.e., higher-order areas) are the first to show age-related decline.
