# Supplementary material for: Cortical similarity networks in the rat brain: Postnatal development and sensitivity to early life stress
Source: Netw Neurosci. 2026 Apr 22;10(2):418–43. doi: 10.1162/NETN.a.546 (PMC13108504; doi:10.1162/NETN.a.546)
Supplement: Supplementary file 1 [file netn-10-2-418-s001.pdf]

Supplemental Material for

Cortical similarity networks in the rat brain: Postnatal development and sensitivity to early life stress

Rachel L. Smith, Stephen J. Sawiak, Lena Dorfschmidt, Ethan G. Dutcher, Jolyon A. Jones, Joel D. Hahn, Olaf Sporns, Larry W. Swanson, Paul A. Taylor, Daniel R. Glen, Jeffrey W. Dalley, Francis J. McMahon, Armin Raznahan, Petra E. Vértes*, Edward T. Bullmore*

Corresponding Author: Rachel L. Smith (rachel.smith5@pennmedicine.upenn.edu)

This PDF file includes

- Supplemental text
- Figures S1 to S6
- Legends for Tables S1 to S3
- SI References

Other supporting materials for this manuscript include the following

- Tables S1-3

Supplemental text

Choice of structural features in MIND networks

The original MIND framework incorporated multiple structural features (e.g., cortical thickness, mean curvature, sulcal depth, surface area, gray matter volume) and employed a k-nearest neighbor approach to estimate symmetric multivariate KL divergence (Sebenius et al. 2023). In the present work, we used only magnetization transfer ratio (MTR) as the input feature. The rationale for this choice was threefold:

First, MTR is widely considered a proxy for myelination, which is of particular relevance in this study as the myelinating process is developmentally dynamic (Hamano et al., 1998; Downes and Mullins, 2014; Mengler et al., 2014) and sensitive to early life stress (Bass et al., 1970; Krigman and Hogan, 1976; Breton et al., 2021; Long et al., 2021; Han et al., 2022; Abraham et al., 2023).

Second, focusing on a single input feature enhances the interpretability of downstream network measures. For example, an increase in edge weight directly indicates greater similarity between the MTR profiles of two regions, which likely reflects increased myelo-architectonic similarity (whereas a decreased edge weight would indicate decreased myelo-architectonic similarity, or increased differentiation between regions). As such, this univariate approach also facilitated more direct comparison to gold-standard tract-tracing data on myelo-architectonic connectivity.

Third, several morphometric features used in the original human MIND implementation are not applicable to the rat brain. Most notably, rats lack cortical folding, and thus measures such as mean curvature and sulcal depth cannot be derived.

Figure S1.

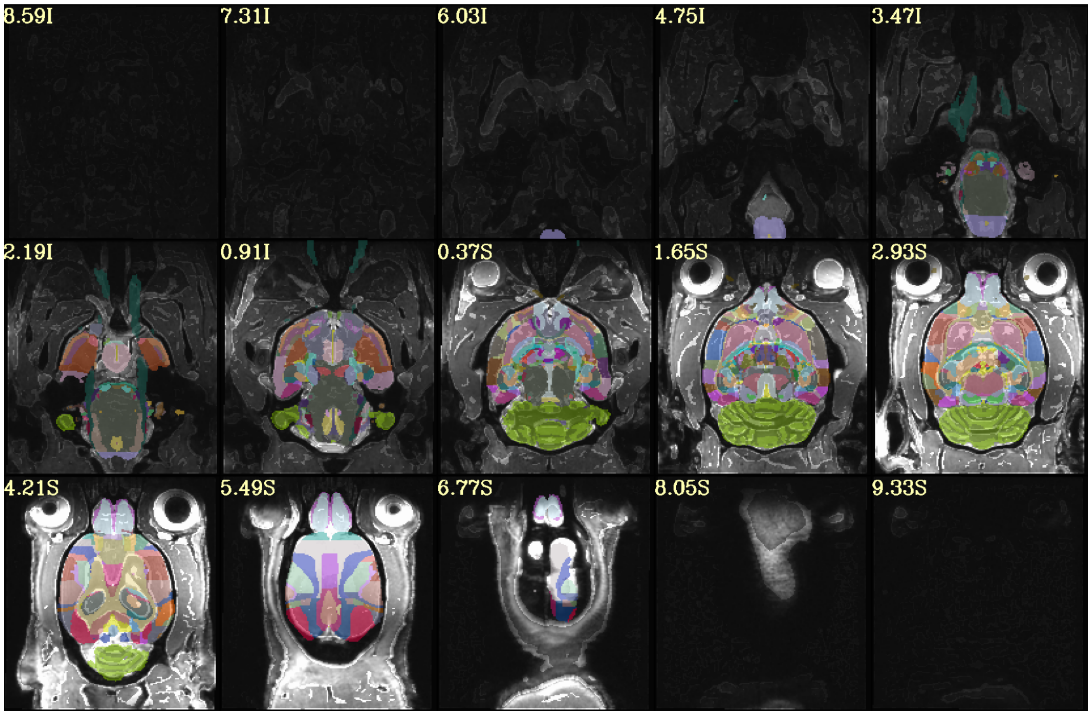

Example quality control images in the axial plane; direct output from the AFNI @animal_warper function. The pipeline also provides quality control (QC) images in the coronal and sagittal slices. The example subject is a post-natal day (PND) 63 rat from the normative developmental cohort.

Figure S2.

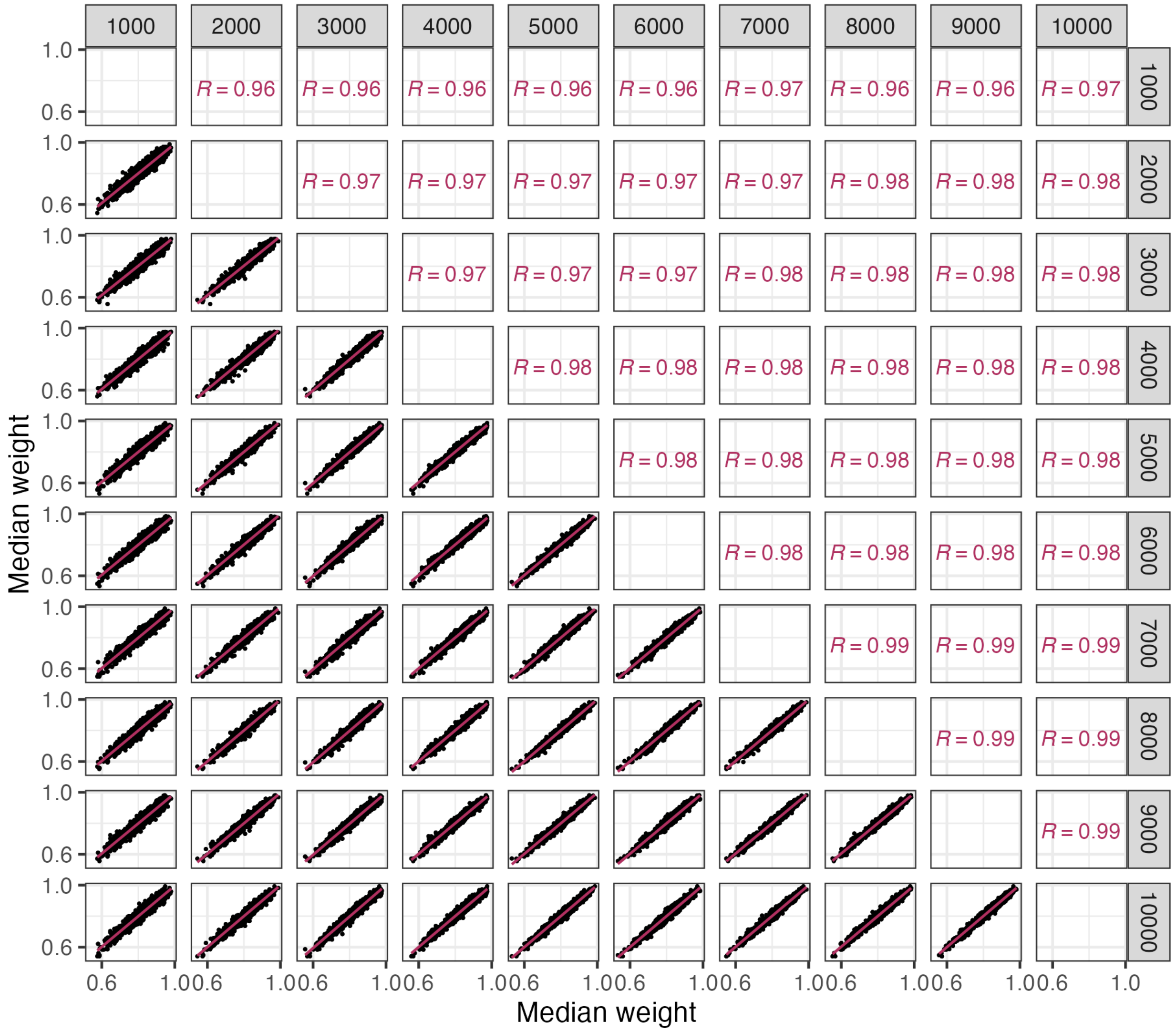

Correlation of all normative networks constructed across different resample values used in network construction. Each column and row represents the normative network constructed from N number of resamples, and the intersecting panel is the comparison between the two networks. The bottom triangle shows the scatterplot, where each point represents an edge. Its x-axis value is the edge weight in the column resample value network, while its y-axis value is the edge weight in the row resample value network. The upper triangle shows the Pearson correlation value between the two networks.

Figure S3.

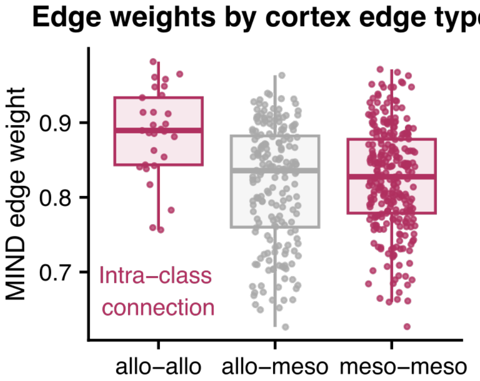

Distributions of intra-cortex class and inter-cortex class morphometric inverse divergence (MIND) edge weights. Maroon indicates a within cortex type connection, while gray indicates a between cortex type connection (allo = allocortex, meso = mesocortex). Eulaminate (isocortical) areas were not considered in this analysis due to too few representative regions.

Figure S4.

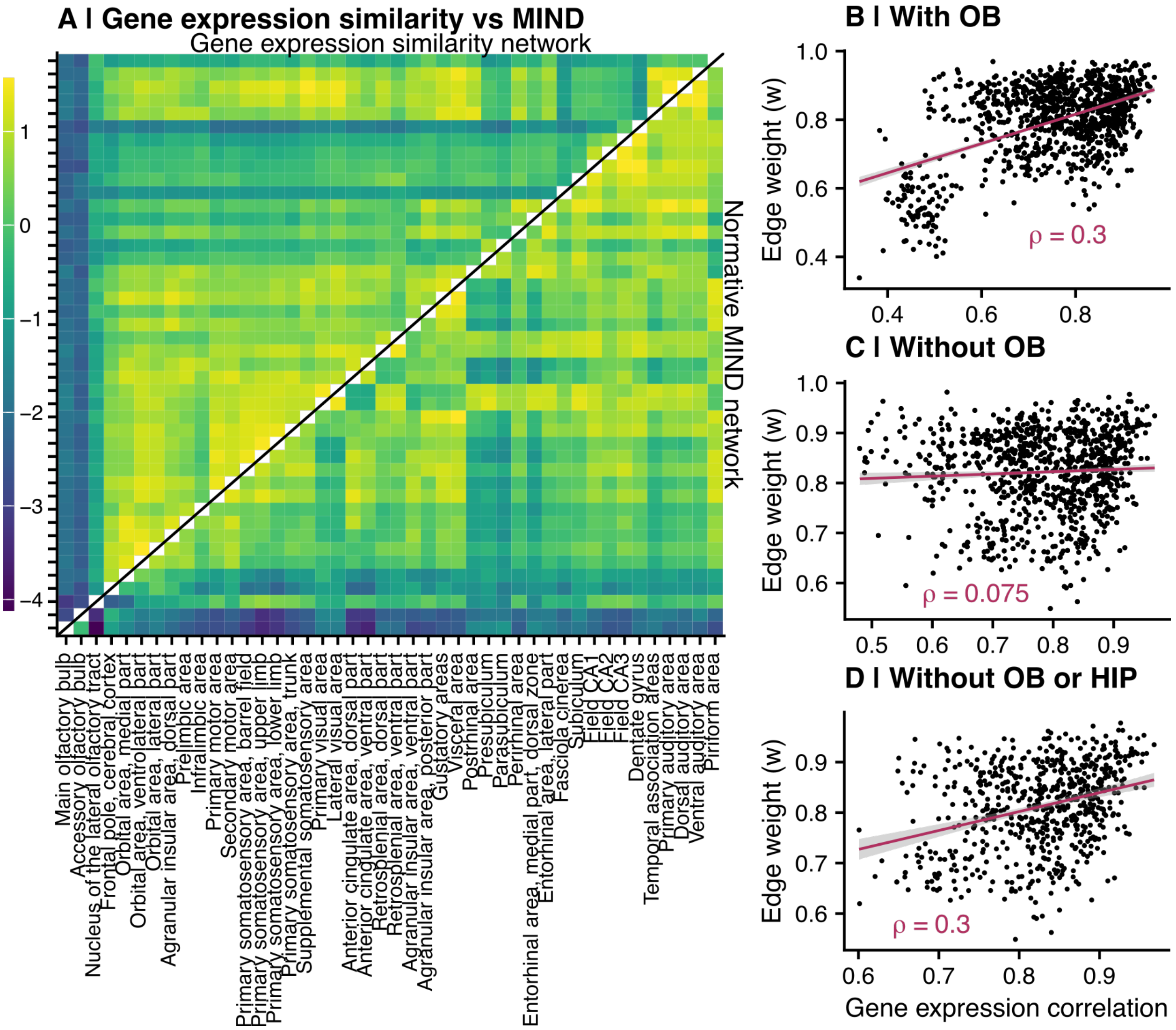

The normative MIND network aligns with spatial gene expression similarity defined in the mouse brain. A) Heatmap representations of the mouse gene expression similarity network (top left of the diagonal) and weighted MIND similarity network (bottom right of the diagonal). Both networks include olfactory bulb (OB) regions in this visualization. Each row and column represent a region of interest, defined by the AMBA (Lein et al., 2007). To increase comparability between the networks, weights were *Z*-scored. **B)** Correlation between mouse gene expression similarity (x-axis) and normative MIND edge weight (y-

axis) when olfactory bulb (OB) regions are included. Each point represents an edge; the line of best fit and Spearman correlation are shown in maroon. **C)** Same as panel B but without any OB regions. **D)** Edges with low similarity in mouse gene expression profiles, but high MIND similarity, principally comprising hippocampal regions, with removal of these edges improving the correlation between MIND and gene expression similarity (ρ=0.32).

Figure S5.

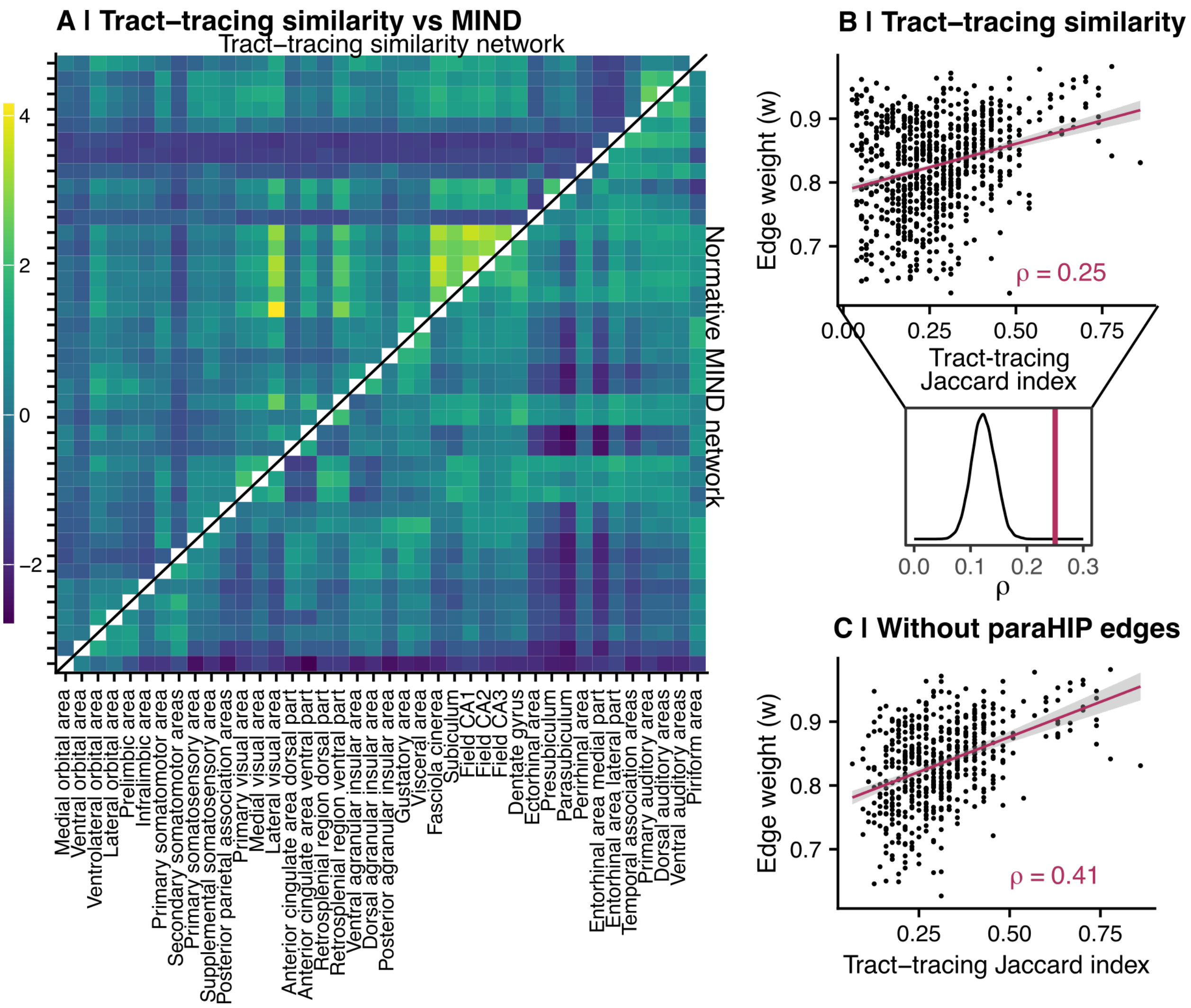

The normative MIND network reflects similarity of axonal connectivity. A) Heatmap representations of the tract-tracing similarity network (top left of the diagonal) and weighted MIND similarity network (bottom right of the diagonal). Each row and column represent a region of interest, defined by the Brain Maps 4.0 atlas (Swanson, 2018). To increase comparability between the networks, weights were *Z*-scored. **B)** Top: Correlation between similarity of tract-tracing connection profiles between pairwise combinations of regions and strength of MIND similarity (same as **Figure 4D**). Bottom: This relationship was significant compared to a null distribution of 10000 distance-corrected networks (normative network Spearman correlation *Z*=6;

$P<0.001$). **C)** Edges with low similarity in tract-tracing profiles, but high MIND similarity, principally comprised parahippocampal regions, with removal of these edges improving the correlation between MIND and tract-tracing similarity (ρ=0.41).

Figure S6.

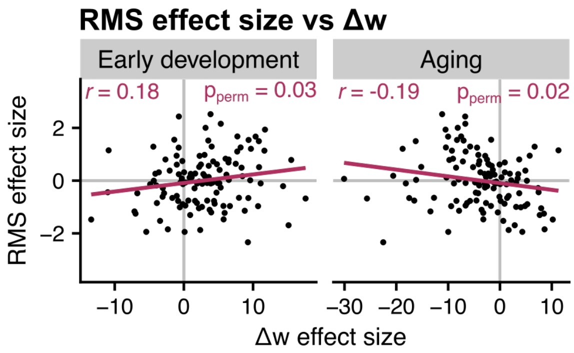

The relationship between normative developmental change and repeated maternal separation (RMS)-induced network perturbations in early adulthood. The x-axis represents Δ*w* from the normative developmental cohort (left = early development; right = aging), while the y-axis shows the PND 63 RMS-control effect size from the experimental cohort. Each point represents a system-level edge, while the line of best fit is shown in maroon. RMS edge assignments were permuted 10000 times, and the actual Pearson correlation was scaled in relation to the null distribution. RMS effect size was significantly associated with both Δw_{dev} (r=0.18, P_{perm}=0.03, Z_{perm}=1.90) and Δw_{age} (r=-0.19, P_{perm}=0.02, Z_{perm}=-2.03).

Legends for Supplemental Tables S1 and S2

Table S1 (separate file). Anatomical correspondence of the Waxholm Space Atlas to other rat brain atlases, including:

A. Brain Maps 4 (Swanson, 2018)

B. Zilles atlas (Zilles, 2012)

C. Allen Mouse Brain Atlas (Lein et al., 2007)

Atlas mappings were generated using the methodology described in the **Methods** section.

Table S2. Comparison of Pearson's (*r*) and Spearman's (*ρ*) correlation coefficients for all tested relationships. In the main text, Pearson's was used for continuous, approximately linear relationships; Spearman's for rank-ordered or non-linear relationships. Results were concordant across methods.

Table S3 (separate file). The {53×53} matrix representation of the normative cortical MIND network, defined as the median edge weight across N=41 individuals at the PND 63 timepoint in the normative developmental cohort.

SI References

Lein ES et al. (2007) Genome-wide atlas of gene expression in the adult mouse brain. Nature 445:168–176.

Swanson LW (2018) Brain maps 4.0—Structure of the rat brain: An open access atlas with global nervous system nomenclature ontology and flatmaps. J Comp Neurol 526:935–943.

Zilles K (2012) The Cortex of the Rat: A Stereotaxic Atlas. Springer Science & Business Media.
